# A simple cost-effective high performance liquid chromatographic assay of sulphadoxine in whole blood spotted on filter paper for field studies

**DOI:** 10.1186/1475-2875-8-238

**Published:** 2009-10-24

**Authors:** Grace O Gbotosho, Christian T Happi, Abayomi O Sijuade, Akin Sowunmi, Ayoade MJ Oduola

**Affiliations:** 1 Department of Pharmacology and Therapeutics, College of Medicine, University of Ibadan, Ibadan, Nigeria; 2 Malaria Research Laboratories, Institute for Advanced Medical Research and Training, College of Medicine, University of Ibadan, Ibadan, Nigeria; 3 WHO/TDR, Geneva, Switzerland

## Abstract

**Background:**

Artesunate plus sulphadoxine-pyrimethamine is one of the four artemisinin-based combination therapies currently recommended by WHO as first-line treatment for falciparum malaria. Sulphadoxine-pyrimethamine is also used for intermittent preventive treatment for malaria in pregnancy. Drug use patterns and drug pharmacokinetics are important factors impacting the spread of drug resistant parasites hence it is imperative to monitor the effect of pharmacokinetic variability on therapeutic efficacy. Unfortunately, information on the pharmacokinetics of sulphadoxine in children and pregnant women with malaria is very limited. Methods for the assay of sulphadoxine-pyrimethamine have been previously reported, but they are not cost-effective and practicable in analytical laboratories in low resource areas where malaria is endemic. Efforts in this study were thus devoted to development and evaluation of a simple, cost-effective and sensitive method for quantification of sulphadoxine in small capillary samples of whole blood dried on filter paper.

**Methods:**

Sulphadoxine was determined in whole blood by reversed-phase high performance liquid chromatography with UV detection at 340 nm. Sulisoxazole (SLX) was used as internal standard. Chromatographic separation was achieved using a Beckman Coulter ODS C_18 _and a mobile phase consisting of 0.05 M phosphate buffer-methanol-acetonitrile (70:17:13 V/V/V) containing 1% triethylamine solution.

**Results:**

Standard curves from sulphadoxine-spiked blood added to filter paper were linear over the concentration range studied. Linear regression analysis yielded correlation coefficient r^2 ^> 0.99 (n = 6). Extraction recoveries were about 82-85%. The limit of quantification was 120 ng/ml while the within and between assay coefficient of variations were < 10%. The inter-day precision was < 5.8% and inter-day accuracy ranged from 4.1 to 5.3%. There was no interference from endogenous compounds or any of the commonly used anti-malarial, analgesic and anti-infective drugs with the peaks of SDX or the internal standard.

**Conclusion:**

The recovery and accuracy of determination of SDX from whole blood filter paper samples using the method described in this study is satisfactory, thus making the method a valuable tool in epidemiological studies and therapeutic drug monitoring in developing endemic countries. Furthermore, the applicability of the method in studying the pharmacokinetic disposition of SDX in a patient suggests that the method is suitable in malaria endemic areas.

## Background

Malaria remains a major public health problem in disease endemic areas affecting mostly young children and pregnant women. Presently, the artemisinin-based combination therapy (ACT) is now recommended as first line treatment for falciparum malaria [1]. Artesunate plus sulphadoxine-pyrimethamine is one of the four ACT currently recommended by WHO [1] for definitive treatment of malaria, while sulphadoxine-pyrimethamine is also used for intermittent preventive treatment for malaria in pregnancy (IPTp) [2] and in infants (IPTPi) [3]. In areas where sulphadoxine-pyrimethamine remains effective, the combination of artesunate with sulphadoxine-pyrimethamine enhances the clinical outcome.

Drug use patterns and drug pharmacokinetics are some of the factors which impact the spread of drug resistant parasites hence the importance of monitoring the effect of pharmacokinetic variability on therapeutic efficacy cannot be overemphasized. Information on the pharmacokinetics of sulphadoxine in children and pregnant women with malaria is very limited. Thus simple, cost effective and sensitive methods for quantification of sulphadoxine and pyrimethamine in small capillary samples of biological fluids dried on filter are necessary.

Although, methods for the assay of sulphadoxine-pyrimethamine have been previously reported, they are not cost effective requiring the use of large volume of blood/plasma, freezing facilities or expensive instrumentation, such as solid phase extraction, photodiode detector or mass spectrophotometer [4-6]. These methods may not be very practicable in analytical laboratories in low resource areas where malaria is endemic. In addition, some of the previous methods used sulphamethoxazole as internal standard [7]. This compound is a component of co-trimoxazole, which is commonly used to treat bacterial infections in some countries including Nigeria and which could interfere with analysis. Presented in this report, is the development and evaluation of a simple, cost-effective two-step extraction procedure, using a reverse-phased chromatographic system with UV detection for the assay of sulphadoxine in whole blood using filter paper. This method would be useful in epidemiological and pharmacokinetic studies in malaria endemic countries where resources are scarce.

## Methods

### Chemicals

Sulphadoxine (Hoffman La Roche, Basel, Switzerland) and sulisoxazole were obtained from Dr. Dennis Kyle, Walter Reed Army Research Institute, USA. Sulisoxazole was used as internal standard. All solvents used were HPLC grade (Merck, D-6100 Darmstadt, Germany) while hydrochloric acid, phosphoric acid and all other reagents employed were analytical grade BDH (Poole, UK). Sulphadoxine-pyrimethamine tablets (Fansidar^®^, Swiss Pharma Nigeria Ltd, under license of F. Hoffmann-La Roche, Basel Switzerland) were purchased locally from a wholesale Pharmacy in Ibadan, Nigeria. Filter papers (Whatman^®^, Maidstone, UK) were also purchased locally.

### Instrumentation

The separation of sulphadoxine was carried out under isocratic condition at room temperature. The HPLC system consisted of a Cecil 4100 pump), attached to Cecil 4200 variable wavelength UV-Visible detector set at 340 nm. The chromatograms were recorded and analyzed with PowerStream software (CE 4900) provided with the instrument (Cecil Instrument, Cambridge, England). Chromatographic separation was achieved using a reversed-phased Ultrasphere ODS 15 cm × 4.6 mm I.D 5 μm C_18 _column (Beckman, USA) maintained at room temperature. The mobile phase consisted of 0.05 M phosphate buffer-methanol-acetonitrile (70:17:13 V/V/V) containing 1% triethylamine solution and adjusted to pH 3.4 with phosphoric acid. Elution was carried out at room temperature and the flow rate was 0.9 ml/min. A syringe loading sample injector model 7725 (Rheodyne L.P., California, USA) was used for sample injection onto the HPLC column.

### Calibration and sample preparation

Stock solutions of sulphadoxine (SDX) (1 mg/ml) and the internal standard, sulisoxazole (SLX) (1 mg/ml) was prepared in 70% methanol. Working solutions of the drugs were prepared by appropriate serial dilution in 0.1 N HCl to yield 100 μg/ml and 500 μg/ml of SDX and SLX respectively. Drug free human whole blood was spiked with the working solution of SDX to yield final concentrations of 100, 80, 40, 20, 10, 5, and 0 μg/ml of sulphadoxine. The resulting solutions were used to develop and evaluate the method. Using an adjustable pipette, 100 μl of spiked whole blood was adsorbed onto Whatman filter paper. The samples were dried at room temperature overnight. Filter papers were stored in desiccated resealable plastic bags at room temperature if they were not to be assayed immediately.

### Extraction procedure

The dried drug-spiked spot of whole blood on filter paper were cut into small pieces. All the pieces from each sample were transferred into clean 15 ml polypropylene centrifuge tubes containing twenty microlitre (20 μl) of 500 μg/ml SLX as internal standard (IS) and 200 μl 0.1 NHCl and soaked for 30 minutes. The extraction solvent acetonitrile (2 ml) was added and the mixture was vortexed for two minutes and thereafter centrifuged for 10 minutes at 2000 g. The supernatant was transferred into a clean centrifuge tube and dried under a stream of nitrogen gas in a water bath at 50°C. The sample was reconstituted in one hundred microlitres (100 μl) of 0.1 N HCl, vortexed for 2 minutes and an aliquot (20 μl) was injected onto the column.

### Precision, accuracy and recovery

Sample preparation and extraction were performed on six replicates at each concentration of sulphadoxine on each of six days. Calibration curves were prepared from the measurement of peak height ratios of the analytes and internal standard. To assess precision and reproducibility of the method, coefficients of variation (CVs) and standard deviations were determined for intra- and inter-assay variability. Recovery was determined for each concentration by comparison of peak height ratio of the extracted known standards with the directly injected standard concentrations.

### Interference

Commonly used anti-malarial, analgesic and anti-infective drugs including chloroquine, mefloquine, quinine, amodiaquine, paracetamol, sulphamethoxazole and trimethoprim were studied for interference by spiking the drugs in blank whole blood. The drugs were extracted according to the method described above. The presence of peaks was monitored after injection of 20 ul of the reconstituted sample.

### Stability

The stability of sulphadoxine blood samples dried on filter paper samples has been previously reported [7].

### Clinical evaluation

In order to evaluate the clinical application of this method, one hundred microlitre (100 μl) of capillary blood obtained from a patient with acute uncomplicated *Plasmodium falciparum *malaria who had taken a standard single oral dose of 25 mg/kg sulphadoxine-pyrimethamine (based on the sulphadoxine component) was spotted on filter paper. Whole blood samples were taken on days 0 before drug administration and days 1, 2, 3, 4, 5, 6, 7, 14 and 28 after drug administration following verbal informed consent from patient's guardian. Samples were dried and extracted as described above.

## Results

### Evaluation of the analytical procedure

**Figure 1:**
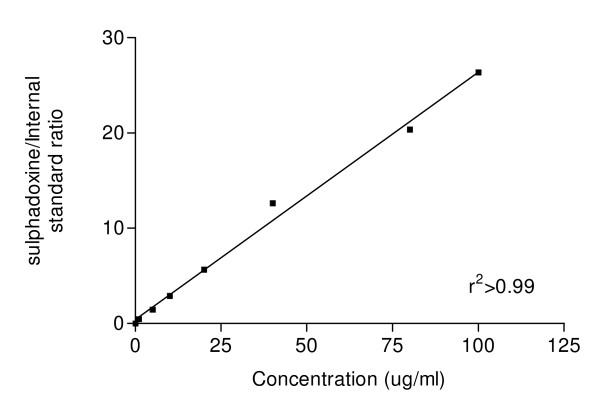
**Calibration curve for sulphadoxine in whole blood**.

**Figure 2:**
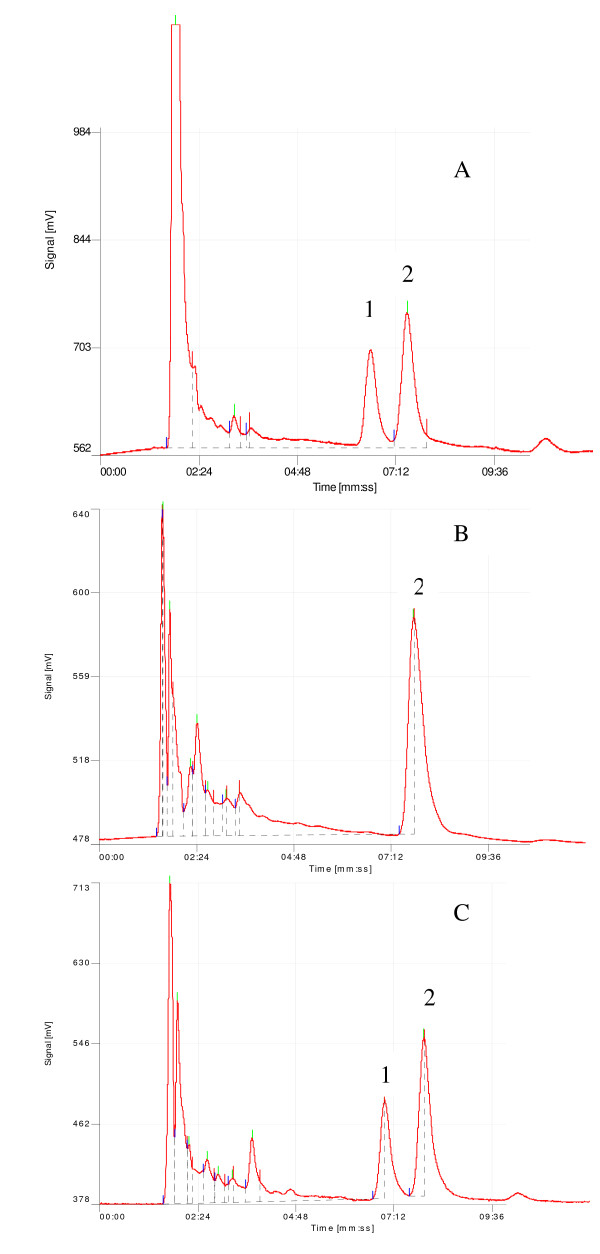
**(A) separation of sulphadoxine (1) and internal standard (2) from whole blood spiked with 60 μg/ml sulphadoxine**. (B) and (C) chromatograms of day 0 and day 3 whole blood samples collected from a patient who received a standard single dose of sulphadoxine-pyrimethamine (25 mg/kg body weight of sulphadoxine component and 1.25 mg/kg body weight of pyrimethamine component).

Standard curves from sulphadoxine-spiked blood added to filter paper were linear over the concentration range studied. Linear regression analysis yielded correlation coefficient r^2 ^> 0.99 (n = 6) (Figure 1). The baseline of sulphadoxine and the internal standard were well resolved at the calibration ranges of 5 - 100 μg/ml with retention times of 6.3 and 7.2 min for sulphadoxine and sulisoxazole respectively (Figure 2A). The separation chromatograms of sulphadoxine and the internal standard from spiked whole blood samples (Figure 2A) corresponded with those of blood samples obtained from a patient at time 0 before drug administration and day 3 after an oral standard dose of sulphadoxine-pyrimethamine (25 mg/kg body weight of sulphadoxine component and 1.25 mg/kg body weight pyrimethamine component) (Figure 2B and 2C respectively).

### Recovery, calibration curves and reproducibility

The extraction recoveries for 25 μg/ml, 60 μg/ml and 100 μg/ml of sulphadoxine were 82.66% ± 4.09 (n = 6), 81.02% ± 3.24 (n = 5) and 85.60% ± 1.98 (n = 5) respectively. The intra-day recovery deviation at 60 μg/ml and 100 μg/ml of SDX were 3.7% and 4.6% respectively (n = 5). The inter-day precision was < 5.8% and inter-day accuracy ranged from 4.1 to 5.3%. The limit of detection was 120 ng/ml at 0.05 absorbance units full-scale (aufs).

### Interference

There was no interference from endogenous compounds or any of the commonly used anti-malarial, analgesic and anti-infective drugs with the peaks of SDX or the internal standard.

### Clinical application

**Figure 3:**
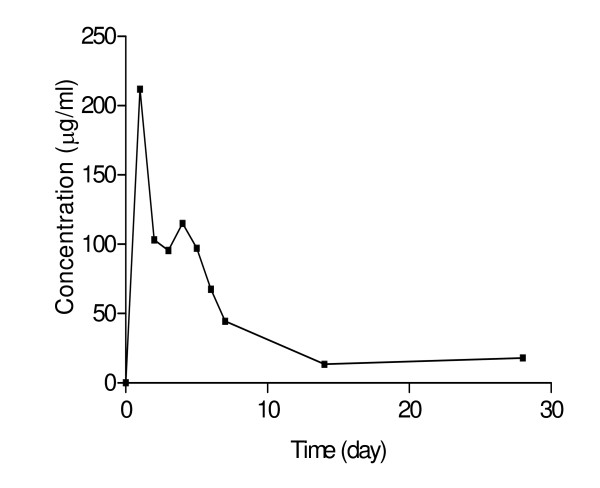
**Pharmacokinetic profile of sulphadoxine in whole blood after administration of single oral dose of sulphadoxine-pyrimethamine (25 mg/kg body weight of sulphadoxine and 1.25 mg/kg body weight of pyrimethamine)**.

The concentration-time profile of SDX in whole blood in the individual who took a standard single oral dose of sulphadoxine-pyrimethamine (SP) (25 mg/kg body weight of sulphadoxine and 1.25 mg/kg body weight pyrimethamine) is shown in Figure 3. The pharmacokinetic parameters of SDX in the individual are shown in table 1. Peak blood concentration was 212.02 μg/ml. The calculated elimination half-life was 3.50 days while the area under the concentration time curve (AUC_0-28_) was 884.84 μg/ml.day. The profile shows the applicability of the method for measuring SDX in whole blood dried on filter paper.

**Table 1:** Pharmacokinetic parameters of sulphadoxine in a patient with *falciparum *malaria treated with a single standard oral dose of sulphadoxine-pyrimethamine tablet.

**Parameter**	
T _1/2 _(day)	3.50

C_max _(μg/ml)	212.02

AUC_0-28 _(μg/ml.day)	884.84

AUC_0-28_: Area under the blood concentration-time curve from time 0 to day 28

C_max_: Peak blood concentration

T_1/2_: Elimination half life

## Discussion

Filter paper methods are suitable for field use because they require small volume of blood samples and no freezing facilities. The method described in this report involves two simple extraction steps followed by drying, reconstitution and injection into the chromatographic system. The recovery and accuracy of determination of SDX from whole blood filter paper samples using this method is satisfactory thus making the method a valuable tool in epidemiological studies and therapeutic drug monitoring in developing endemic countries. Recovery may however have been better without affecting cost of analysis, if the extraction had been performed with two aliquots of one millilitre each of acetonitrile as opposed to one aliquot of two millilitres of acetonitrile. The applicability of the method in studying the pharmacokinetic disposition of SDX in a patient suggests that the method is suitable in malaria endemic areas. In this method, sulisoxazole was used as internal standard. Sulisoxazole is not a commonly used sulpha drug in this area, thus it is very convenient in determination methods because there is rare likelihood of interference with drugs previously taken by patients or volunteers. However, a limitation of this method is that it did not detect pyrimethamine in whole blood samples although the pyrimethamine peak was separated from the sulphadoxine peak in spiked whole blood samples. Thus there will not be any interference of peaks during analysis. Lack of detection of pyrimethamine in patient samples may be attributable to the wavelength for detection not being optimal for pyrimethamine. Previous studies have however shown that knowledge of the SDX concentration provides some information on the magnitude of the accompanying PYR concentrations [7].

The present method can be used in conjunction with the simple pyrimethamine method described by Minzi [8] to study sulphadoxine-pyrimethamine drug combination. The present method is simple, cost effective and practical in resource poor settings compared to previously reported methods which require use of larger volumes of blood, freezing facilities or expensive instrumentation [4-6], although it requires a relatively time consuming organic solvent evaporation step. It would be useful in monitoring sulphadoxine level following IPT in infants (IPTi) or pregnant women (IPTp) with sulphadoxine-pyrimethamine or during treatment of falciparum malaria with artesunate-sulphadoxine/pyrimethamine or any combination therapy that may involve a sulfa-based compound for malaria treatment especially in resource poor malaria endemic areas.

## Conclusion

The applicability of this method in studying pharmacokinetic disposition of sulphadoxine in a patient suggests that the method is suitable in malaria endemic areas. In addition the ability to quantify sulphadoxine in whole blood using a sensitive and cost-effective assay makes the method useful in epidemiological studies and therapeutic drug monitoring in low resource settings.

## Competing interests

The authors declare that they have no competing interests.

## Authors' contributions

GOG was responsible for conceptualization and design of the study, developing the research protocol, data analysis and manuscript preparation. CH participated in study design and protocol development and manuscript preparation. AOS participated in study design, protocol development and data analysis and manuscript preparation. AS participated in design and clinical evaluation of method. AMJO participated in conceptualization of the study.
